# Therapeutic vaccine to cure large mouse hepatocellular carcinomas

**DOI:** 10.18632/oncotarget.19367

**Published:** 2017-07-18

**Authors:** Zhen Han, De Yang, Anna Trivett, Joost J. Oppenheim

**Affiliations:** ^1^ Cancer and Inflammation Program, Center for Cancer Research, National Cancer Institute at Frederick, Frederick, MD, USA

**Keywords:** HMGN1, R848, anti-PD-L1, anti-CTLA4, cytoxan

## Abstract

Hepatocellular carcinoma (HCC) is one of the most common malignancies worldwide with limited therapeutic options. Here we report the development of a therapeutic vaccination regimen (shortened as ‘TheraVac’) consisting of intratumoral delivery of high-mobility group nucleosome-binding protein 1 (HMGN1), R848/resiquimod, and one of the checkpoint inhibitors (e.g. anti-CTLA4, anti-PD-L1, or low dose of Cytoxan). C57BL/6 mice harboring large (approximately 1 cm in diameter) established subcutaneous Hepa1-6 hepatomas were cured by intratumoral injections of TheraVac and became tumor-free long-term survivors. Importantly, the resultant tumor-free mice were resistant to re-challenge with Hepa1-6 hepatoma, not B16 melanoma, demonstrating the acquisition of hepatoma-specific immunity in the absence of any administered tumor antigen. Mechanistic studies showed that upon treatment with TheraVac, Hepa1-6-bearing mice generated increased Hepa1-6-specific CTLs in the draining lymph nodes and showed greatly upregulated expression of CXCL9, CXCL10, and IFN-γ and elevated infiltration of T lymphocytes in tumor tissues. Treatment of large Hepa1-6 hepatomas on one mouse flank also eliminated smaller (approximately 0.5 cm in diameter) hepatomas implanted on the other flank. Thus, TheraVac has potential as a curative immunotherapeutic regimen for the treatment of human HCC.

## INTRODUCTION

Hepatocellular cancer (HCC) remains one of the deadliest cancers since it is refractory to classic chemotherapy and irradiation. Only surgical resection of early lesions offers limited chances for cure [1–4]. Liver transplantation has been used as a treatment for early liver tumors, but recurrence is common and donor organs are scarce [5]. The receptor tyrosine kinase inhibitor (RTKI), Sorafenib, is a currently standard drug for unresectable HCC. However, HCC rapidly becomes sorafenib-resistant. Therefore, more effective therapy is required for HCC.

Over the past decade, tremendous progress has been made in the development of immunotherapies for cancer. Checkpoint inhibitors such as antibodies against cytotoxic T-lymphocyte-associated protein 4 (anti-CTLA4) and programmed cell death 1 (anti-PD-1), etc, have been shown to be partially effective against several malignant tumors [6, 7] including HCC [8]. Cytotoxic T-lymphocyte (CTL) response has been positively linked to anti-tumor immune responses [9, 10]. However, more than 96% tumor-infiltrating lymphocytes (TILs) in HCC have been demonstrated to be quiescent [11], without cytotoxic effect on tumor cells. Therefore, combination of immunotherapeutic agents inducing active effector T cells and blocking elements that negatively regulate T-cell responses may represent an effective strategy to treat HCC [12, 13].

Generation of effector tumor-specific CTL can be enhanced by the use of various adjuvants such as damage-associated molecular patterns (DAMPs), alarmins, pathogen-associated molecular patterns (PAMPs), and proper delivery systems (e.g. virosome, immunostimulating complexes, etc) capable of promoting antigen uptake by dendritic cells (DCs) [14–20]. Most DAMPs and PAMPs trigger pattern recognition receptors (PRRs), including Toll-like receptors (TLRs) [16, 19, 21]. TLR activation initiates divergent signaling pathways, which exert either a pro- or an anti-tumor response [22]. Hypoxia may induce the translocation of an alarmin, HMGB1, from the nucleus to the cytosol, which forms complexes with mitochondrial DNA and mediates tumor growth of HCC by stimulating TLR9 [23]. In contrast, TLR4 contributes to the generation of anticancer immune responses following chemotherapy, radiotherapy, or administration of another alarmin, high-mobility group nucleosome binding protein 1 (HMGN 1) [20]. HMGN1 is a TLR4 ligand that enhances Th1 immune responses and induces prophylactic anti-tumor immunity [14, 24]. HMGN1 is effective in preventing the growth of mouse thymoma and melanoma [14]. Since hmgn1^−/−^ mice spontaneously develop tumors, are more susceptible to induced carcinogenesis and show reduced resistance to thymoma implantation [14, 25], we hypothesized that HMGN1 may participate in the induction of immune responses against a variety of tumors. In addition to TLR4 ligands, TLR7/8 agonists such as R848 (Resiquimod) have also been demonstrated to promote antitumor immune responses [26, 27]. A related TLR7 agonist, imiquimod, has been used for the topical treatment of various epidermal malignancies [27, 28].

In this study, we investigated whether combinations of TLR stimulants inducing antitumor CTLs together with checkpoint inhibitors overcoming suppression of T cell responses might provide more effective HCC treatment using mouse ectopic Hepa1-6 cells as a model which was originally considered non-immunogenic [29]. Our results show that HMGN1 and R848 induce CTLs, while inhibiting immunoregulatory elements [30–32] were blocked by antibodies against checkpoint inhibitor (e.g. anti-CTLA4, anti-PD1/anti-PD-L1) or by Cytoxan, which at low doses reduces the number and function of regulatory T cells [16, 33, 34]. Consequently, C57BL/6 mice bearing large (around 1 cm diameter) Hepa1-6 tumors were cured with the combination of HMGN1, R848, and one of the checkpoint inhibitors such as anti-CTLA4, anti-PD-L1 or Cytoxan. The tumor free mice were resistant to subsequent re-challenge with Hepa1-6 hepatoma, but not unrelated tumors, indicative of persistent of tumor-specific immunity. Thus, effective anti-tumor responses were generated in mice against an HCC model originally believed to be non-immunogenic.

## RESULTS

### Mice bearing large Hepa1-6 tumors were cured by a combination of HMGN1, R848 and anti-PD-L1 and developed tumor-specific immunity

**Figure 1:**
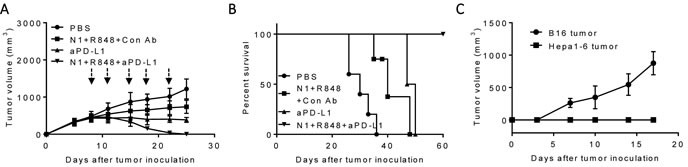
Antitumor effect of a triple combination regimen (HMGN1, R848 and anti-PD-L1) on mice harboring large Hepa1-6 tumors C57BL/6 mice (female, 8 weeks old, *n* = 5) inoculated *s.c*. with 2×10^6^/mouse of Hepa1-6 cells were treated with four i.t. injections of HMGN1 (N1), R848 and anti-PD-L1 (all 10 mg/injection/tumor) on day 8, 11, 15, 18 and 22 (blue arrows) in various combinations. Tumor formation and growth were monitored as indicated and tumor size was plotted. The mice were monitored for tumor growth **A**., and survival **B**. All five mice treated with the triple combination of HMGN1, R848, and anti-PD-L1 became tumor-free, which, after eight weeks, were inoculated *s.c*. with 2×10^5^/mouse of B16F10 cells and 2×10^6^/mouse of Hepa1-6 cells in contralateral flanks. The formation and growth of B16 melanoma and Hepa1-6 hepatoma were monitored and plotted **C**.

A therapeutic vaccination regimen termed “TheraVac” was developed by employing two strategies simultaneously: one aimed at activating antitumor immune responses; the other was to block one of the checkpoint inhibitors so that the immune responses induced would not be dampened. For the activation of anti-Hepa1-6 immune responses, the combination of HMGN1, a TLR4 agonistic alarmin, and R848/resiquimod, a ligand for TLR7/8 were used since HMGN1 and R848 were found to synergistically promote IL-12 production by DCs *in vitro* (unpublish observation). C57BL/6 mice were subcutaneously inoculated with syngeneic Hepa1-6 tumor cells in the right flank to form solid tumors. Since large experimental tumors are likely to be more representative of advanced human HCC, Hepa1-6 tumors were allowed to grow in mice until approximately 1 cm in diameter before the initiation of treatment with biweekly intratumoral (*i.t*.) administration of HMGN1 and R848 in the absence or presence of anti PD-L1 antibody. As shown in Figure 1A, treatment with HMGN1 (10 mg/mouse) and R848 (10 mg/mouse) by *i.t*. injection slowed the growth rate of subcutaneous Hepa1-6 tumors in mice. Intratumoral treatment with anti-PD-L1 (10 mg/mouse) also reduced the further growth of Hepa1-6 tumors. However, both approaches alone did not result in any shrinkage of existing Hepa1-6 tumors. Nevertheless, combination of HMGN1, R848 with anti-PD-L1 after *i.t*. injection initially halted Hepa1-6 tumor growth, followed a week later by the shrinkage of existing Hepa1-6 tumors. The established Hepa1-6 tumors disappeared after five TheraVac treatments. Hepa1-6 tumor-bearing mice treated with the triple TheraVac became tumor-free for up to eight weeks (Figure 1B). When Hepa1-6 tumor-free mice were inoculated *s.c*. with Hepa1-6 cells into the right flank, and B16 melanoma cells into the left flank, all mice grew solid B16 tumors on the left flank, whereas none of the mice grew Hepa1-6 tumors on the right flank. These results indicate that cured mice acquired Hepa1-6 tumor-specific immunity (Figure 1C).

### Treatment with TheraVac consisting of HMGN1, R848 and anti-PD-L1 markedly increased the infiltration of tumors by activated effector T cells

**Figure 2:**
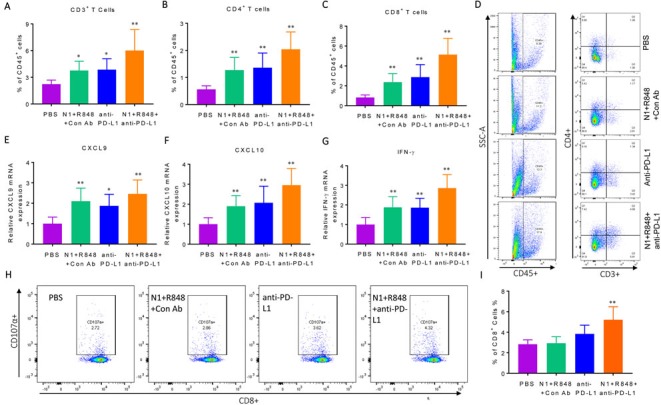
Immunological profiling of Hepa1-6 tumor-bearing mice with various treatments Four groups of C57BL/6 mice (female, 8 weeks old, *n* = 5) were inoculated s.c. with 2×10^6^/mouse of Hepa1-6 cells in the right flank on day 1 and the treatment started on day 8 with two i.t. injections of N1, R848, or anti-PD-L1 (all at 10 mg/injection/tumor) in various combinations on day 8, 12, and 15. Forty-eight hours after the last treatment, the residual tumors were resected flow cytometry analysis of lymphocyte infiltration or RNA extraction. A-C, infiltration of lymphocyte was plotted as the average (mean ± SD) of CD3^+^ **A**., CD4^+^ **B**. and CD8^+^ **C**. T cells in each group, *p<0.05. Dot plot of one representative mouse of each group is shown **D**. The results of one experiment representative of three are illustrated. **E**.-**G**., the expression of CXCL9 (E), CXCL10 (F) and IFN-γ (G) mRNA was graphed as the average (mean ± SD) of three experiments. **H**., **I**. Hepa1-6 bearing C57BL/6 mice (female, 8 weeks old, *n* = 3) were prepared and treated as in A-F. Lymphocytes in the draining inguinal lymph nodes of each group used for the measurement of Hepa1-6-specific CTLs using the CD107a mobilization assay. A representative dot plot of one mouse from each group is shown (H) and statistical analysis is provided (I).

To gain insight into the mechanistic basis for C57BL/6 mice bearing Hepa1-6 tumors to respond to TheraVac treatment, residual tumors were resected *en masse* two days after the third treatment to obtain single tumor cell suspension for analysis by flow cytometry. While treatment with HMGN1 plus R848 or anti-PD-L1 alone caused significant increase in total CD3^+^ T cells, as well as CD4^+^ T and CD8^+^ T subsets in the tumors, treatment with the triple TheraVac resulted in the highest infiltration by T cells (Figure 2A-2D). qPCR revealed that the triple treatment resulted in the highest expression of mRNA for the T cell specific chemokines CXCL9 and CXCL10 in tumors, suggesting that these two chemokines might be responsible for the enhanced infiltration of CD4^+^ and CD8^+^ T cells in the tumor tissues after treatment (Figure 2E-2F). In addition, triple TheraVac also induced the highest-level expression of IFN-g mRNA in the tumor tissues (Figure 2G).

To ascertain that Hepa1-6 tumor specific CTLs were induced, lymphocytes in the tumor-draining LNs were incubated together with Hepa1-6 cells *in vitro* for 24 h to determine the percentage of CD107a^+^ CD8 T cells. CD107a transiently appears onto the cell surface when CTLs degranulate upon encountering target cells [37], which serves as an indicator of the cytotoxic activity of CTLs [36]. As shown in Figure 2H, 2I, after TheraVac treatment the draining LNs of Hepa1-6-bearing mice contained the highest percentage of CD107a^+^ CD8^+^ T cells (as compared with PBS treatment). Therefore, the curative effect of TheraVac on large Hepa1-6 tumors was associated with the induction of effector CTLs, presumably recruited into the tumors by CXCL9 and CXCL10 chemokines.

### TheraVac regimen consisting of HMGN1, R848 and anti-CTLA4 also cured large Hepa1-6 tumors in mice

**Figure 3:**
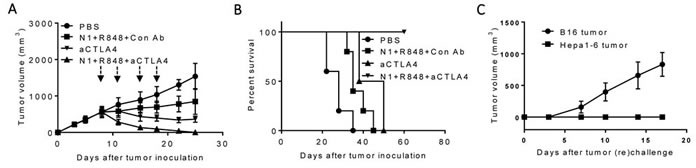
Antitumor effect of a triple combination of HMGN1 (N1), R848 and anti-CTLA4 on mice harboring big Hepa1-6 tumors C57BL/6 mice (female, 8 weeks old, *n* = 5) were inoculated *s.c*. with 2×10^6^/mouse of Hepa1-6 cells in the right flank on day 1 and the formation of tumors were monitored. When tumors reached approximately 1 cm in diameter (day 8), the mice treated with one dose of i.t. anti-CTLA4 (10 mg/injection/tumor) on day 8 (blue arrow) and/or four i.t. injections of N1 (10 mg/injection/tumor) and R848 (10 mg/injection/tumor) on day 8, 11, 15, and 18 (black arrows). Tumor growth (**A**., mean ± SD) and survival **B**., were monitored and plotted (purple circle = PBS, green circle = N1 + R848 + Control-Ab, blue circle = anti-CTLA4, orange circle = N1 + R848 + anti-CTLA4). The results of one experiment representative of three is shown. **C**. Hepa1-6-free mice resulting from treatment with the combination of N1+ R848+ anti-CTLA4 in B were *s.c*. inoculated with 2×10^6^/mouse of Hepa1-6 cells in the right flank and 2×10^5^/mouse of B16F10 cells in the left flank. The appearance and growth of solid tumors were monitored for up to five weeks.

We next investigated whether anti-PD-L1 antibody in the regimen could be replaced by another checkpoint inhibitor, anti-CTLA4 antibody. As shown in Figure 3A, *i.t*. doses of H MGN1 (10 mg/mouse) plus R848 (10 mg/mouse) biweekly for two weeks significantly reduced, and together with anti-CTLA4 (10 mg/mouse) halted, the growth of existing large tumors in mice. Combination of HMGN1, R848 and anti-CTLA4 resulted in the shrinkage and disappearance of large preexisting Hepa1-6 tumors. Hepa1-6-bearing mice treated with the triple TheraVac remained tumor-free for up to 8 weeks (Figure 3B). Challenge of tumor free mice with *s.c*. inoculation of Hepa1-6 and B16F10 cells into the contralateral flank revealed that all mice grew B16 tumors, whereas none of the mice grew Hepa1-6 tumors (Figure 3C). These results indicate the acquisition of Hepa1-6-specific immune memory by the cured mice despite the fact that no exogenous Hepa1-6-associated tumor-associated antigen (TAAs) were administered.

**Figure 4:**
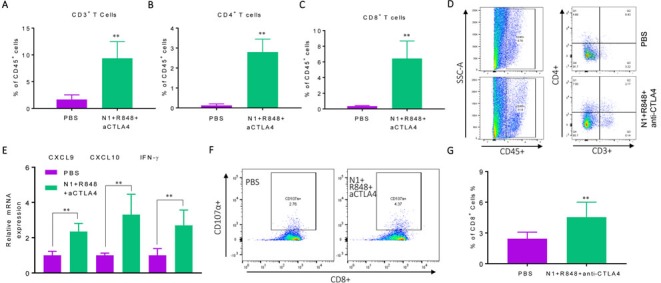
Hepa1-6-bearing mice treated with triple combination of HMGN1, R848, and anti-CTLA4 exhibited elevated antitumor immune responses C57BL/6 mice (female, 8 weeks old, *n* = 5) were inoculated *s.c*. with 2×10^6^/mouse of Hepa1-6 cells in the right flank on day 1 were treated starting on day 8 with three rounds of i.t. injections of either PBS or PBS containing N1, R848, and anti-CTLA4 (all at 10 mg/injection/tumor) on day 8, 12, and 15. Forty-eight hours after the last treatment, the residual tumors were resected for the preparation of single cell suspension or RNA extraction. The single cell suspensions were analyzed by flow cytometry after immunostaining. The average (mean ± SD) of CD3^+^ **A**., CD4^+^ **B**., CD8^+^ **C**. T cells in each group were plotted. *p<0.05. Dot plot of one representative mouse of each group is shown **D**. The result of one experiment representative of three is shown. The RNA preparation were used for the quantitation of CXCL9, CXCL10 and IFN-γ **E**. mRNA levels by qPCR. The average (mean ± SD) of three experiments is shown. **F**., **G**. Hepa1-6 bearing C57BL/6 mice (female, 8 weeks old, *n* = 3) were prepared and treated as in A-F. The draining inguinal lymph nodes were removed 48h after the last treatment to make single cell suspensions for the measurement of Hepa1-6-spacific CTLs using the CD107a mobilization assay. Dot plot of one representative mouse of each group is shown (F) and statistical analysis is provided (G).

Hepa1-6 tumor bearing mice after three rounds of treatment with HMGN1, R848 and anti-CTLA4 showed significantly increased infiltration of CD3^+^, CD4^+^, and CD8^+^ T cells (Figure 4A-4D) in tumor tissues. Additionally, the expression levels of mRNA for IFN-γ, CXCL9 and CXCL10 were significantly elevated in the tumor tissues (Figure 4E). Measurement of CD107a^+^ CD8 T cells showed considerably higher (*p*< 0.001) percentage of CTLs in the draining LNs of treated mice (Figure 4F, 4G). Therefore, anti-CTLA4 can replace anti-PD-L1 in the triple regimen to cure mice bearing large Hepa1-6 tumors with the induction of tumor-specific immune responses.

### Cytoxan (CY) replaced checkpoint inhibitor antibodies in the TheraVac regimen to eliminate large Hepa1-6 tumors

**Figure 5:**
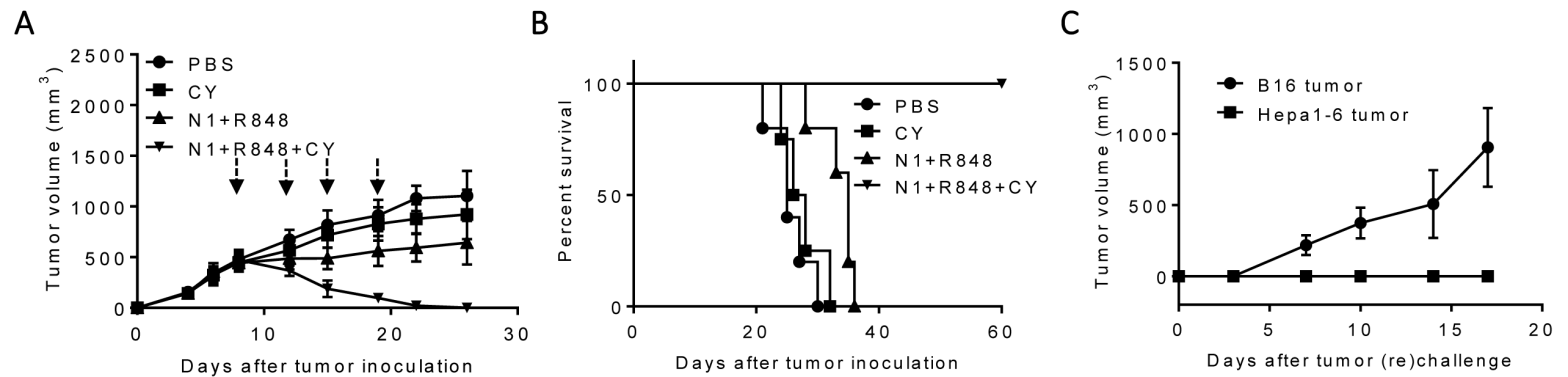
The antitumor effect of triple combination of HMGN1, R848 and low dose CY on large Hepa1-6 tumors C57BL/6 mice (female, 8 weeks old, *n* = 5) were inoculated *s.c*. with 0.2 ml PBS containing 2×10^6^ Heap1-6 cells in one flank on day 1. After tumors reached approximately 1 cm in diameter on day 8, mice were injected *i.t*. with 0.1 ml PBS or PBS containing N1, R848, and one dose of Cytoxan *i.p*. (CY, 2 mg/mouse, blue arrow) on day 8, 12, 15, and 19. The mice were monitored for tumor growth **A**. and survival **B**. Eight weeks after the treated mice became tumor-free, the tumor-free were *s.c*. inoculated with identical number (2×10^6^/mouse) of Hepa1-6 and B16F10 (2×10^5^/mouse) melanoma cells in contralateral flanks and monitored for the formation and growth of Hepa1-6 hepatoma and B16 melanoma **C**. All data are representative of at least two experiments.

We then investigated the possibility to replace checkpoint inhibitor antibodies with Cytoxan (CY) in treating large Hepa1-6 hepatoma in mice, since CY at low doses mimics checkpoint inhibitors and suppresses Tregs [16, 33–34]. C57BL/6 mice bearing large Hepa1-6 tumors were treated once with a low dose of intraperitoneal (i.p.) CY (2 mg/mouse) or i.t. injection of HMGN1 and R848 (10 μg/mouse/injection) alone or in combination. As anticipated, CY alone slightly reduced the rate of growth of CT26 Hepa1-6 tumors, while four rounds of treatment with HMGN1 and R848 showed more significant inhibition of tumor growth (Figure 5A). Four rounds of *i.t*. treatment with R848 (10 μg/mouse/injection), HMGN1 (10 μg/mouse/injection), and one *i.p*. injection of CY completely eradicated existing tumors in mice within two weeks (Figure 5A). Hepa1-6 tumor bearing mice treated with the triple combination showed long-term tumor-free survival (Figure 5B). When such mice were re-challenged *s.c*. with Hepa1-6 cells into the right flank and B16 melanoma cells into the left flank, all mice grew B16 tumors, whereas no Hepa1-6 tumor appeared on the right flank, indicating that vaccinated mice acquired tumor-specific immunity (Figure 5C). Therefore, Cytoxan can replace anti-CTLA 4 or anti-PD-L1 in the triple regimen to eliminate large established hepatomas in mice.

### TheraVac was effective for the treatment of metastatic Hepa1-6 tumors

**Figure 6:**
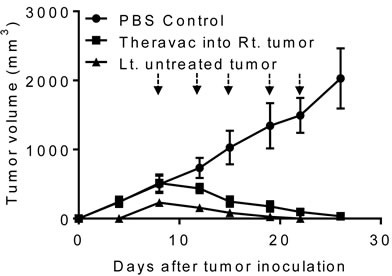
The antitumor effect of triple combination regimen consisting of N1, R848, and a checkpoint inhibitor on metastasis of Hepa1-6 tumors Two groups (G1-G2) of female C57BL/6 mice (8 weeks old, *n* = 5) were inoculated *s.c*. with 2×10^6^/mouse of Hepa1-6 in the right flank on day 1. On day 5, mice in G2 were inoculated *s.c*. with 2×10^6^/mouse of Hepa1-6 cells in the left flank. Mice were treated with PBS (G1) or a combination (G2) of one *i.p*. Cytoxan (CY, 2 mg/mouse, blue arrow) and four *i.t*. injections of N1 and R848 (each at 10 μg/injection/tumor, black arrows) into the right tumors twice weekly starting on day 12. Tumor growth (mean ± SD) was monitored and plotted (Rt. and Lt. = right and left tumor, respectively; untreated tumor: non-HMGN1 and R848 treated tumor).

To simulate cancer metastasis, C57BL/6 mice were inoculated *s.c*. with Hepa1-6 into the right flank, and 4 days later inoculated with Hepa1-6 into the left flank. By day 8, mice

grew large Hepa1-6 tumors in the right flank and small tumors in the left flank. Hepa1-6 tumor bearing mice were then treated with i.t. injection of HMGN1 and R848 into the larger tumors on the right flank and one dose of intraperitoneal (*i.p*.) CY (2 mg/mouse) injection for the subsequent two weeks (Figure 6). As a result, treatment with the triple regimen eradicated large tumors established on the right flank of mice. The treatment also caused complete regression of smaller Hepa1-6 tumors on the left flank, suggesting that the triple therapeutic regimen was effective in eradicating lesions at a distant site. Thus, the triple therapeutic regimen induced systemic antitumor immunity. However, if tumors on the left flank grew to the size equal (1 cm in diameter) to the tumors on the right flank, TheraVac *i.t*. in tumors on the right flank only moderately reduced the growth of the tumors on the left flank, suggesting the treatment was less effective systemically than locally.

## DISCUSSION

Numerous approaches have been tried previously to develop better therapies for HCC. One approach was to combine vaccination with irradiated GM-CSF-expressing tumor cells and low dose CY. Although this promoted anti-Hepa1-6 tumor immune responses and markedly inhibited the growth of subcutaneous Hepa1-6 tumors, it did not eradicate Hepa1-6 tumors in mice even when the treatment began on day 3 after Hepa1-6 cell inoculation [31]. A combination of polyI:C and Sorafenib was found to significantly limit the growth of subcutaneous Hepa1-6 tumors, but also failed to completely eliminate the tumors [30]. Treatment of ectopic hepatoma with a combination of DCs and HCC-specific Listeria vaccine capable of triggering both TLR4 and NLRP3 inflammasome pathways, or CD40L-expressing DCs, only reduced, but did not eliminate tumors [32, 37]. Other studies have tried to treat mouse experimental hepatomas using α-fetoprotein DNA vaccination combined with i.t. administration of AdmIL-12 and AdmIP-10 [38], a combination of oral Sunitinib (a small multi-targeted RTK inhibitor) and adoptive transfer of hepatoma-specific CTLs [39], or combined irradiation of tumors and AdmFlit3L/AdmCD40L [40, 41]. However, none was capable of eliminating large hepatomas. Therefore, TheraVac appears to be the first immunotherapeutic approach that consistently eradicated large experimental hepatomas.

Our HCC therapeutic regimen consisted of two arms: one was to promote antitumor immune host responses using a combination of HMGN1 and R848; the other aimed to disarm immunosuppression, by using checkpoint inhibitors anti-PD-L1 and anti-CTLA4 or low dose of CY to reduce Treg function. The triple therapeutic regimen not only eradicated large established solid Hepa1-6 hepatomas, but also induced tumor-specific immunity, because the tumor free mice became selectively resistant to rechallenge with Hepa1-6 cells (Figures 1, 3, & 5). Treatment with this regimen resulted in the acquisition of specific protective immunity against secondary Hepa1-6 hepatomas without administration of any exogenous TAAs, presumably due to the release of endogenous tumor antigen captured by DCs which were locally activated by *i.t*. TheraVac. This outcome resembled successful vaccination of treated tumor(s) and we therefore proposed the name TheraVac as a short form of ‘Therapeutic vaccination’. It should also be noted that the mice bearing a large tumor were cured within 14 days after initiation of TheraVac therapy. Such a rapid response may be attributable to an expansion of preexisting immunity, which is enhanced by the checkpoint inhibitors contained in TheraVac.

HMGN1 and R848 have been shown to act as immune stimulants and promote Th1 immune response [24, 42], which mediates protection against tumors. Indeed, Hepa1-6 tumor bearing mice treated with a combination of HMGN1 and R848 showed significant retardation of tumor growth (Figures 1, 3, & 5). In addition, treatment with HMGN1 and R848 increased CTLs in the draining LNs and upregulated chemokines CXCL9 and CXCL10, as well as IFN-γ, all indicators of Th1 immunity in the tumor tissue. These chemokines may recruit activated CD4 and CD8 lymphocytes into tumors (Figures 2 & 4). However, immunosuppressive microenvironment in tumors often nullify antitumor immune responses [15–16, 43]. It has been reported that the numbers of Tregs, MDSC, PD-1^+^ exhausted T cells, and levels of immunosuppressive cytokines are all increased in patients with hepatocellular carcinoma, compared with normal controls, revealing a network of potential mechanisms of immune dysregulation in patients with hepatocellular carcinoma” [15, 16]. Therefore, inclusion of a checkpoint inhibitor (e.g. anti-PD-L1, anti-CTLA4) or CY in the regimen substantially improved the therapeutic effectiveness that completely eradicated transplanted HCC (Figures 1, 3, & 5).

Although the pharmacokinetics of our TheraVac requires further investigation, the doses and time points of administration we used were effective. In addition, TheraVac shows unique advantages: First, TheraVac has little side effect, since it does not inhibit the proliferation of tumor cells in vitro, nor does it require systemic administration. Mice harboring large Hepa1-6 hepatomas treated with TheraVac did not experience significant loss of body weight or patchy hair loss (data not shown). Additionally, TheraVac achieved successful hepatoma therapy without using exogenous TAAs, thus, bypassing the necessity for the identification of TAAs. Finally, all the components of TheraVac have no obvious antigenicity and are less likely to induce immune reactions on repeated usage. HMGN1 is a protein normally present in mammalian cells, while anti-PD-L1 and anti-CTLA4 are humanized and have already been approved for clinical use. R848 is a small synthetic derivative of imidazoquinonine and CY at low suboptimal doses selectively inhibits regulatory T cells. The only disadvantage is the need to deliver R848 and HMGN1 into the tumor tissues, which may not be easily translated into use on inaccessible tumors. We are currently investigating the means of overcoming this limitation by using nanoparticles for the delivery of R848 and HMGN1 into tumor tissues upon systemic administration [44].

## MATERIALS AND METHODS

### Cell lines, mice and reagent

All cell lines [Hepa1-6 hepatoma cell line (CRL-1830) and B16F10 melanoma cell line (CLR-6475)] used in the present study were initially purchased from the American Type Culture Collection (ATCC, Manassas, VA). Hepa1-6 hepatocellular carcinoma and B16F10 melanoma cells were maintained in DMEM medium (Meditech, Manassas, VA) supplemented with 10% FBS (Hyclone, South Logan, UT), 2 mmol/L-glutamine, 25 mmol/L HEPES, 100 U/mL penicillin,100 mg/mL streptomycin]. C57BL/6 mice (8 - 12 weeks old, female) were provided by the Animal Production Area of the National Cancer Institute (NCI) at Frederick, MD. NCI-Frederick is accredited by AAALAC International and follows the Public Health Service Policy for the Care and Use of Laboratory Animals. Animal care was provided in accordance with the procedures outlined in the “Guide for Care and Use of Laboratory Press” (Washington, D.C.). Animal studies were approved by the Institutional Animal Care and Use Committee (IACUC) of NCI at Frederick (Frederick, MD). Recombinant HMGN1 was produced in-house using Baculovirus expression system in insects as previously reported [14]. R848, anti-PD-L1 (clone 10F.9G2) and anti-CTLA4 (clone BE0131) were purchased from Bio X Cell (West Lebanon, NH, USA) and Cytoxan was purchased from Sigma-Aldrich (St. Louis, MO, USA).

### Mouse tumor model and treatment

Female mice (C57BL/6, *n* = 5-10, 8-12-week old) were subcutaneously injected with 0.1 ml PBS containing Hepa1-6 (2×10^7^/ml) or B16F10 (2×10^6^/ml) into their flank (left or right) regions. The appearance and size of tumors as well as the mouse body weight were monitored twice weekly. The length (L) and width (W) of tumors were measured with a caliper. Tumor size was calculated by the formula: (L×W^2^)/2. When tumors reached approximately 1 cm in diameter, the tumor-bearing mice were treated with intraperitoneal (*i.p*.) injection of Cytoxan (2 mg/0.2 ml/mouse), intratumoral (i.t.) injection of anti-CTLA4 (10 μg/0.05 ml/mouse), anti-PD-L1 (10 μg/0.05 ml/mouse), i.t. injection of HMGN1 (10 μg/0.05ml/mouse), and *i.t*. injection of R848 (10 μg/0.05ml/mouse) alone or in combination as specified using PBS as a negative control. To determine whether mice cured of Hepa1-6 tumors acquired tumor-specific immune protection, tumor-free mice were re-challenged with Hepa1-6 and B16F10 (used as unrelated tumors) cells on contralateral flanks and the formation and growths of tumor were monitored. In certain experiments, Hepa1-6 tumors were resected and used for the isolation of RNA or measurement of leukocyte infiltration.

### Collection of tumors and draining lymph nodes

Residual tumors and draining lymph nodes (LN) were resected, weighed, and sliced into approximately 1 mm^3^ cubes in cold Leibovitz L-15 medium (Meditech). Draining lymph nodes (LNs) were weighed and cut into pieces in L-15 medium. Both tumors and draining LNs were subsequently digested for two rounds in Leibovitz L-15 medium (at 25 ml/gram) containing a cocktail of enzymes, each at 37°C for 45 minutes with constant slow shaking (∼80 rpm), with vigorous pipetting in-between. The enzymatic cocktail consisted of 0.17 mg/ml collagenase I (Worthington Biochemical Corp., Lakewood, NJ), 0.056 mg/ml collagenase II (Worthington Biochemical), 0.17 mg/ml collagenase VI (Worthington Biochemical), 0.025 mg/ml deoxyribonuclease I (Worthington Biochemical), and 0.025 mg/ml elastase (Worthington Biochemical) as previously reported [45].

The final mixtures were passed through 70-mm cell strainers, washed three times with PBS, and suspended in PBS to make single-cell suspensions.

### Quantitative real-time polymerase chain reaction (qRT-PCR)

Total RNA was extracted using the Qiagen RNeasy Mini Kit, according to the manufacturer's protocol (Qiagen, Germantown, MD). RNA was quantified using a NanoDrop 1000 UV-Vis spectrophotometer (Thermo Scientific, Wilmington, DE, USA). Single-strand cDNA was synthesized using 1 μg of total RNA using a high-capacity cDNA kit (Qiagen). The primer sequences were: mouse IFN-γ [PPM03121A-200, Reference position 291 (K00083)], mouse β-actin [PPM02945B-200, Reference position 533 (NM_007393)], mouse CXCL9 [PPM02973B-200, Reference position 2619 (NM_008599)] and mouse CXCL10 [PPM02978E-200, Reference position 633 (NM_021274)]. The annealing temperature was 52°C for IFN-γ, CXCL9, and CXCL10, and 60°C for β-actin. Real-time RT-PCR data were analyzed using the ^ΔΔ^CT method. Data are expressed as mean ± standard error, where each treatment was performed in triplicate.

### CD107a mobilization assay

Mobilization of CD107a onto the surface of CTLs as a result of activation-induced degranulation was used to as an indicator of the cytotoxic function of Hepa1-6-specific CTLs [46].

Draining LNs were resected and digested as mentioned above to make a single-cell suspension. Hepa1-6 cells (2×10^5^/ml) seeded in wells of a 24-well plate as target cells and then lymphocytes from draining LNs (1×10^6^/ml) were added at an effector to target ratio (E: T) of 5:1 incubated at 37°C in a humid atmosphere (5% CO_2_) for 24 h to induce granule exocytosis. Lymphocytes incubated without Hepa1-6 cells served as a negative control. Subsequently, the lymphocytes were harvested, washed with FACS buffer (PBS containing 0.5% BSA and 0.05% NaN_3_), blocked with 2% normal mouse serum on ice for 10 minutes and stained with PE-Cy5-anti-mouse CD8a (BD/Pharmingen, clone 53-6.7) and eFlour 660-anti-CD107α (eBioscience, clone 1D4B) antibodies on ice for 30 minutes. The samples were resuspended in FACS buffer and data were acquired on a LSRII flow cytometer (BD).

### Immunostaining and flow cytometry

Single cells of dissociated mouse Hepa1-6 tumors were suspended in FACS buffer, blocked with 2% normal mouse serum on ice for 10 minutes and stained with various combinations of fluorophore-conjugated anti-mouse antibodies on ice for 30 minutes. The antibodies used to stain the samples were PerCP-Cy 5.5-anti-mouse CD45 (TONBO Bioscience, clone 104), FITC-anti-mouse CD3 (TONBO Bioscience, clone 17A2), PE-Cy5 anti-mouse CD8a (BD/Pharmingen, clone 53-6.7) and APC-anti-mouse CD4 (TONBO Bioscience, clone GK 1.5). The stained samples were analyzed and collected on a LSRII flow cytometer (BD). All flow cytometry data were analyzed using FlowJo.

### Statistical analysis

Unless otherwise specified, all experiments were performed at least three times, and the results of representative experiments or the means of the data from multiple experiments are shown. Differences in the *in vivo* tumor growth were determined by repeated ANOVA measures, whereas differences between the control and experimentally treated mouse groups were evaluated by one-way ANOVA after arcsine square-root transformation.

## Abbreviations

HCC: Hepatocellular carcinoma
TheraVac: Therapeutic vaccination
HMGN1: High-mobility group nucleosome-binding protein 1
CY: Cytoxan
RTKI: Receptor tyrosine kinase inhibitor
FDA: The Food and Drug Administration
CTLA4: Cytotoxic T-lymphocyte-associated protein 4
PD-1: Programmed cell death 1
CTL: Cytotoxic T-lymphocyte
TILs: Tumor-infiltrating lymphocytes
DAMPs: Damage-associated molecular patterns
PAMPs: Pathogen-associated molecular patterns
DCs: Dendritic cells
PRRs: Pattern recognition receptors
TLRs: Toll-like receptors
SDS-PAGE: Sodium dodecyl sulfate polyacrylamide gel electrophoresis
